# An abscisic acid (ABA) homeostasis regulated by its production, catabolism and transport in peanut leaves in response to drought stress

**DOI:** 10.1371/journal.pone.0213963

**Published:** 2019-06-26

**Authors:** Haitao Long, Zhao Zheng, Yajun Zhang, Pengzhan Xing, Xiaorong Wan, Yixiong Zheng, Ling Li

**Affiliations:** 1 School of Life Sciences, South China Normal University, Guangzhou, China; 2 College of Agriculture and Biology, Zhongkai University of Agriculture and Engineering, Guangzhou, China; Key Laboratory of Horticultural Plant Biology (MOE), CHINA

## Abstract

ABA is an important messenger that acts as a signaling mediator for regulating the adaptive response of plants to drought stress. Two production pathways, *de novo* biosynthesis and hydrolysis of glucose-conjugated ABA by β-glucosidase (BG), increase cellular ABA levels in plants. ABA catabolism via hydroxylation by 8’-hydroxylase (CYP707A), or conjugation by uridine diphosphate glucosyltransferase (UGT), decreases cellular ABA levels. The transport of ABA through ATP-binding cassette (ABC)-containing transporter proteins, members of ABC transporter G family (ABCG), across plasma membrane (PM) is another important pathway to regulate cellular ABA levels. In this study, based on our previously constructed transcriptome of peanut leaves in response to drought stress, fourteen candidate genes involved in ABA production (including *AhZEP*, *AhNCED1* and *AhNCED3*, *AhABA2*, *AhAAO1* and *AhAAO2*, *AhABA3*, *AhBG11* and *AhBG24*), catabolism (including *AhCYP707A3*, *AhUGT71K1* and *AhUGT73B4*) and transport (including *AhABCG22-1* and *AhABCG22-2*), were identified homologously and phylogenetically, and further analyzed at the transcriptional level by real-time RT-PCR, simultaneously determining ABA levels in peanut leaves in response to drought. The high sequence identity and very similar subcellular localization of the proteins deduced from 14 identified genes involved in ABA production, catabolism and transport with the reported corresponding enzymes in databases suggest their similar roles in regulating cellular ABA levels. The expression analysis showed that the transcripts of *AhZEP*, *AhNCED1*, *AhAAO2* and *AhABA3* instead of *AhABA2*, *AhNCED3* and *AhAAO1* in peanut leaves increased significantly in response to drought stress; and that the *AhBG11* and *AhBG24* mRNA levels were rapidly and significantly up-regulated, with a 4.83- and 4.58-fold increase, respectively at 2-h of drought stress. The genes involved in ABA catabolism *AhCYP707A3*, *AhUGT71K1* instead of *AhUGT73B4* were significantly induced in response to drought stress. The expression of two closely related peanut *ABCG* genes, *AhABCG22*.*1* and *AhABCG22*.*2*, was significantly up-regulated in response to drought stress. The ABA levels rapidly began to accumulate within 2 h (a 56.6-fold increase) from the start of drought stress, and peaked at 10 h of the stress. The highly and rapidly stress up-regulated expressions of genes involved in ABA production and transport, particularly *AhNCED1*, *AhBG11* and *AhBG24*, and *AhABCG22*.*1* and *AhABCG22*.*2*, might contribute to the rapid ABA accumulation in peanut leaves in response to drought. In response to drought stress, ABA accumulation levels in peanut leaves agree well with the up-regulated expressions of ABA-producing genes (*AhZEP*, *AhNCED1*, *AhAAO2*, *AhABA3*, *AhBG11* and *AhBG24*) and PM-localized ABA importer genes (*AhABCG22-1* and *AhABCG22-2*), in spite of the simultaneously induced ABA catabolic genes (*AhCYP707A3* and *AhUGT71K1*), although the induction of catabolic genes was much lower than that of biosynthetic gene (*AhNCED1*). This difference in induction kinetics of gene expression may define the significant accumulation of drought-induced ABA levels. These results suggest that ABA homeostasis in peanut leaves in response to drought maintained through a balance between the production, catabolism and transport, rather than simply by the biosynthesis.

## Introduction

The plant hormone ABA plays pivotal roles in many important physiological processes including stomatal closure, seed dormancy, growth and various abiotic stress responses [1,2]. ABA is mainly produced by the *de novo* biosynthetic pathway through the oxidative cleavage of carotenoids [3]. In this pathway, zeaxanthin epoxidase (ZEP/ABA1) catalyzes the formation of all transviolaxthin from zeaxanthin [4]. Nine *cis*-epoxycarotenoid dioxygenase (NCED) cleaves carotenoids to form xanthoxin [5,6]. Xanthoxin is assumed to be transported from the plastids to the cytosol, although the precise mechanism that mediates this transport is not yet known [2]. The short-chain alcohol dehydrogenase/reductase (SDR/ABA2) converts xanthoxin derived from the cleavage of carotenoids into abscisic aldehyde [7,8], which is finally oxidized into ABA by abscisic aldehyde oxidase (AAO) [9–11]. Aldehyde oxidase requires the molybdenum cofactor sulfurase/ABA3 to produce a functional cofactor for its catalytic activity [12]. All of the steps of ABA *de novo* biosynthesis occur in plastids except for the final two stages, which take place in the cytosol [9–11].

An alternative pathway for producing ABA is via hydrolysis of ABA-glucosyl ester (ABA-GE), which is an inactive glucose-conjugated form of ABA. Intracellular ABA-GE can be hydrolysed by the two β-glucosidase (BG) homologs AtBG1 and AtBG2 in *Arabidopsis* [13,14], which localize to the endoplasmic reticulum (ER) and vacuole, respectively. The single-step reaction of β-glucosidase-regulated hydrolysis of ABA-GE to ABA is an ideal and important way to achieve the rapid increase in ABA contents necessary for plants to meet their physiological needs [14].

ABA catabolism is also a mechanism for regulating ABA levels. In *Arabidopsis* it proceeds mainly via two pathways, namely ABA 8’-hydroxylation catalyzed by ABA 8’-hydroxylase, the cytochrome P450 (CYP) 707A family [15], and ABA conjugation with glucose mediated by glucosyltransferases [16,17]. The 8′-hydroxylation of ABA is mediated by CYP707A family of proteins (CYP707As 1, 2, 3 and 4) in *Arabidopsis* [15]. We previously reported two genes (*AhCYP707A1* and *AhCYP707A2*) encoding ABA 8’-hydroxylase from peanut [18]. The genes *AhCYP707A1* and *AhCYP707A2* were expressed ubiquitously in peanut roots, stems and leaves with different transcript levels, and were modulated osmotically [18]. The different spatial and temporal patterns of expression of four *Arabidopsis* and two peanut *CYP707A* genes, suggesting that each of the gene products may function in different physiological or developmental processes. The expression of all four *Arabidopsis CYP707A* genes was induced by dehydration stress and subsequent rehydration [15,19], which indicates that ABA levels are regulated by a balance between biosynthesis and catabolism, including feedback-induced catabolism. Conjugation of ABA with glucose is catalysed by ABA-uridine diphosphate (UDP) glucosyltransferases (UGTs), which include *Arabidopsis* UGT71B6 and its two closely related homologs, UGT71B7 and UGT71B8 [16,17]. A recent study has shown that UGT71B6, UGT71B7 and UGT71B8 play crucial roles in ABA homeostasis and adaptation to dehydration, osmotic and high-salinity stresses in *Arabidopsis* [17]. ABA catabolic pathways appear to be localized in the cytosol (UGT71Bs) and the ER membrane (CYP707As) [20].

Moreover, ABA and its metabolites are transported between subcellular compartments within a cell as well as between cells [2,20]. For the regulation of endogenous ABA level in plants, it is still crucial to determine how ABA transport is regulated, and whether it is involved in the control of physiological responses. The protonated ABA could be transported from relatively low-pH to high-pH cellular compartments via a passive diffusion that does not require specific transporters [21]. The first step in ABA transport might be ABA export out of cells. ABA is synthesized in the cytosol, where the pH is relative higher than that in the apoplastic space. Therefore a specific transporter may be required for ABA export to the apoplastic space. Recent studies in *Arabidopsis* have identified both ABA exporters and ABA importers localized to the plasma membrane (PM). ABA transporters were first identified in *Arabidopsis*, and they are ATP-binding cassette (ABC)-containing transporter proteins, members of ABC transporter G family [22,23]. AtABCG25, a half-size ABC transporter protein, is responsible for ABA export from vascular tissues in plants [22]; AtABCG40, a full-size ABC transporter, acts as an ABA importer in plant cells [23]. The discovery of *AtABCG25* and *AtABCG40* strongly suggests the existence of an active control of ABA transport between plant cells [22,23]. Kuromori et al [24] presented that *AtABCG22* encodes a half-size ABC transporter with a function related to guard cell responses in *Arabidopsis*. Kang et al [25] have reported that four AtABCG proteins function together to deliver ABA from the endosperm to the embryo in mature imbibed seeds of *Arabidopsis*. AtABCG25 and AtABCG31, localized to the endosperm, export ABA from the endosperm to the embryo, whereas the embryo-localized AtABCG30 and AtABCG40 transport ABA into the embryo [25]. The low-affinity nitrate transporter (NRT1) was also reported to function as an ABA importing transporter (AIT1) [26,27]. Zhang et al [28] showed that AtDTX50 (Detoxification Efflux Carrier 50), a membrane protein in the MATE (Multidrug and Toxic Compound Extrusion) transporter family in *Arabidopsis*, mediated ABA efflux from the cytosol of vascular and guard cells. Recently, we have also isolated an ABA transporter-like 1 gene (*AhATL1*) from peanut plants, which modulated ABA sensitivity through specifically affecting ABA import into cells in transgenic *Arabidopsis* [29]. It appears that multiple types of transporters are involved in ABA transport in plants. Therefore, ABA-specific transporters localized to the plasma membrane also regulate the cellular ABA levels in plant cells.

Drought is one of the major abiotic stresses that limit the growth and production of plants. The mechanisms of drought stress response have been investigated most extensively in *Arabidopsis*, which include ABA-dependent and ABA-independent pathways [1,30,31]; ABA homoeostasis modulated by its production, inactivation, and transport is considered to play vital roles in plant development and stress responses; the transcriptional regulation of genes involved in either ABA production or ABA inactivation is of great importance in ABA homoeostasis [32]. However, our knowledge of the genes involved in regulation of ABA homoeostasis is relatively rare in agricultural crops in response to drought. We have used peanut, an economically important oil and protein rich crop, to address the issue [18,33–42]. In the present study, based on the screening of our previously constructed transcriptome of peanut leaves in response to drought stress [38], we report the identification and expression analysis of genes encoding the enzymes involved in ABA production [including one *ZEP* (*AhZEP*), two *NCED*s (*AhNCED1* and *AhNCED3*), one *ABA2* (*AhABA2*), two *AAO*s (*AhAAO1* and *AhAAO2*), one *ABA3* (*AhABA3*), and two *BG*s (*AhBG11* and *AhBG24*)], catabolism [including one *CYP707A* (*AhCYP707A3*) and two *UGT*s (*AhUGT71K1* and *AhUGT73B4*)], and transport [including two *ABCG*s (*AhABCG22-1* and *AhABCG22-2*), which jointly contribute to the regulation of ABA homeostasis precisely in peanut leaves in response to drought.

## Materials and methods

### Plants and growth conditions

Seeds of peanut (*Arachis hypogaea* L. cv ‘Yueyou 7’) were sown in pots with a potting mixture of vermiculite, perlite and soil (1:1:1), and grown in a growth chamber with 16 h of light from fluorescent and incandescent lamps (200 μmol m^-2^ s^-2^) followed by 8 h of darkness at 28°C [18]. Plants were watered daily with half-strength Murashige and Skoog nutrient solution [43].

### Drought stress treatment of plants

For the treatment of polyethylene glycol (PEG6000)-simulated drought stress, three-leaf-stage (10–15 days after planting) peanut plants were removed from the soil mixture carefully to avoid injury, and then hydroponically grown in a solution containing 20% (W/V) PEG6000 or deionized water as a control for indicated time, respectively. For all treatments, peanut leaves were frozen in liquid nitrogen immediately following the treatments and stored at -80°C until analysis. The entire experiments were biologically repeated at least three times.

### Molecular cloning of genes encoding enzymes involved in ABA biosynthesis, catabolism and transport from peanut

From the constructed transcriptome which contained 47 842 assembled unigenes of three-leaf-stage peanut leaves in response to drought [38], we screened the fragments of genes encoding the enzymes involved in ABA production (including *AhZEP*, *AhNCED1* and *AhNCED3*, *AhABA2*, *AhAAO1* and *AhAAO2*, *AhABA3*, *AhBG11* and *AhBG24*), catabolism (including *AhCYP707A3*, *AhUGT71K1* and *AhUGT73B4*) and transport (including *AhABCG22-1* and *AhABCG22-2*). The missing 5’ and 3’ ends of the screened genes were obtained by rapid amplification of cDNA ends (RACE) using the GeneRacer kit according to the manufacturer’s instructions (ThermoFisher, Shanghai, China). The gene specific primers for 5’ and 3’ RACE of target genes were listed in Table 1. In all cloning experiments, PCR fragments were gel-purified and ligated into the pMD 19-T Vector (TaKaRa, Dalian, China), and confirmed by sequencing from both strands.

**Table 1:** Primer sequences used in the present study.

Primer name	Sequence (5’ to 3’)	Function
3GSP1-outer	AGGAAGAGTTGGAGGAAGG	3’ RACE for *AhZEP*
3GSP1-inner	TCATTCAGAAGATGATGCC	
ORF-ZEP-F	ATGATGCCTATGATGTTGAGTTGG	Amplification of complete ORF of *AhZEP*
ORF-ZEP-R	CTCTAAATTATAGAGAAGATGG	
5GSP2-outer	TCATCGTCGGAACAGAGAGTCGG	5’ RACE for *AhNCED3*
5GSP2-inner	GGAAATACTTCAGGTACGG	
3GSP2-outer	AGGTGGACCTTGTGAGCGG	3’ RACE for *AhNCED3*
3GSP2-inner	GGAGAGAAGGTTCGGCGG	
ORF-NCED3-F	CTCAATGATCATGGCACC	Amplification of complete ORF of *AhNCED3*
ORF-NCED3-R	AGATTAGACGTGGTGGAG	
5GSP3-outer	GGATGTGAAAAATGCGTG	5’ RACE for *AhABA2*
5GSP3-inner	TGCTTTCTCCAATGCCAC	
3GSP3-outer	TGCTGTACTCTTCTTGGC	3’ RACE for *AhABA2*
3GSP3-inner	TTGATGCTTGATGGAGG	
ORF-ABA2-F	CAACATGTCTTCCTCC	Amplification of complete ORF of *AhABA2*
ORF-ABA2-R	ACAACTACATGTGAAGC	
5GSP4-outer	CCATTGAACTTTGATGTCC	5’ RACE for *AhABA3*
5GSP4-inner	CCATTGCAATGTCTCAAAGCC	
3GSP4-outer	TTGGAAGGCATTGAGAAGGG	3’ RACE for *AhABA3*
3GSP4-inner	TTCTGTTCAAGAAGCTGGCCTC	
ORF-ABA3-F	ATGGATGCTGCTAAGCAAG	Amplification of complete ORF of *AhABA3*
ORF-ABA3-R	CTAAATAGACTCTGGATGAACATGC	
5GSP5-outer	GGAATAAGTCTTGACCAAG	5’ RACE for *AhBG11*
5GSP5-inner	TCTGTAGGCATCTAATCCC	
3GSP5-outer	GATATAATTGCTGCTCAGAGGG	3’ RACE for *AhBG11*
3GSP5-inner	GACTTCTTTCTTGGGTGG	
ORF-BG11-F	ATGTGGAAGAAGGGATTTGTTGTGG	Amplification of complete ORF of *AhBG11*
ORF-BG11-R	TTAAATAGCATTGTGCAATAAGGC	
5GSP6-outer	CCTTTAACCATTAAGGATTGTGCTCC	5’ RACE for *AhBG24*
5GSP6-inner	GATATGTACCAGAATGTAGTGG	
3GSP6-outer	ATAAGGAATGGTGTGAGG	3’ RACE for *AhBG24*
3GSP6-inner	AGGTTACTTTGCATGGTC	
ORF-BG24-F	ATGTGGGTTAAGGGTGGTG	Amplification of complete ORF of *AhBG24*
ORF-BG24-R	CTACCATTTTAATGGTGGAG	
5GSP7-outer	CACCTATGTATGGCCAACCC	5’ RACE for *AhCYP707A3*
5GSP7-inner	TGGAGCCAGGTGGGAGTGG	
3GSP7-outer	CAAGAGACTCTAAGAGTTGCATC	3’ RACE for AhCYP707A3
3GSP7-inner	GAAGATGTGGAATATCAAGGG	
ORF-CYP707A3-F	ATGGAACTAAGTACCATG	Amplification of complete ORF of *AhCYP707A3*
ORF-CYP707A3-R	GTTGATATGCTACTTCTTGGG	
3GSP8-outer	CTCTACGTCGTGACCTTC	3’ RACE for *AhUGT71K1*
3GSP8-inner	CATCGACTCCCTCATACCCCAC	
ORF-UGT71K1-F	AGAAAGAAATGGCAGAGG	Amplification of complete ORF of *AhUGT71K1*
ORF-UGT71K1-R	GCAACAATTGTTTTCAGTAGCTACC	
5GSP9-outer	GGCTATGATTGTTGAGGCGG	5’ RACE for *AhUGT73B4*
5GSP9-inner	GCTGTGTCTATCATTGGG	
3GSP9-outer	GAGTGAAGAAGATTGCTGAGAGTGG	3’ RACE for *AhUGT73B4*
3GSP9-inner	GAGCTGTTGAAGAAGGTGG	
ORF-UGT73B4-F	ATGGCAACTGAAACTGGTTTAG	Amplification of complete ORF of *AhUGT73B4*
ORF-UGT73B4-R	CTAATCTAAACTTCGCCATTGC	
5GSP10-outer	AGCTCTCTTTTCCTTTTGCTCC	5’ RACE for *AhABCG22-1*
5GSP10-inner	CTTTGGAAGTCTAAGCCTGGCTGC	
3GSP10-outer	CAGTAGTGACAACAATTCACCAACC	3’ RACE for *AhABCG22-1*
3GSP10-inner	GTTGATCCTTCTTGGAAAAGGGAGC	
ORF-ABCG22-1-F	ATGGAGAAACCAAATTCAACAACCC	Amplification of complete ORF of *AhABCG22-1*
ORF-ABCG22-1-R	TCATGCTCCGGATTGAAGCTTCATCC	
3GSP11-outer	CTTCAGTGATGATGATGATATCCCG	3’ RACE for *AhABCG22-2*
3GSP11-inner	GATATTGAAGCTGGAACTC	
ORF-ABCG22-2-F	ATGGAGAATGGAAACACGTCATCG	Amplification of complete ORF of *AhABCG22-2*
ORF-ABCG22-2-R	GCTCCATATTATGAAACATTCATCCAACGC	
18S-F	ATTCCTAGTAAGCGCGAGTCATCAG	Internal control for Real-time PCR
18S-R	CAATGATCCTTCCGCAGGTTCAC	
ZEP-F	AAGATGAGATGGAACCCTGC	Real-time PCR for *AhZEP*
ZEP-R	TACATACACCGTCACATCC	
NCED1-F	CTTCTTTCGTGTGAGCGAGG	Real-time PCR for *AhNCED1*
NCED1-R	TGCAGAGAGAAACATGAGCC	
NCED3-F	TACTGTACTCCACCACGTC	Real-time PCR for *AhNCED3*
NCED3-R	GAAACCTGTTAGTCTCCC	
ABA2-F	TAATGCTGTACTCTTCTTGGC	Real-time PCR for *AhABA2*
ABA2-R	ACAACTACATGTGAAGC	
AAO1-F	CCACCATTACTTCTAGCAGC	Real-time PCR for *AhAAO1*
AAO1-R	CAAATCTTGAATCCGTTCC	
AAO2-F	CAACACTGAATTGGTTGGTTG	Real-time PCR for *AhAAO2*
AAO2-R	GTATTTTATACTGGTGGCTGG	
ABA3-F	AGTTGACTTATGGGACTC	Real-time PCR for *AhABA3*
ABA3-R	GATGTATAAAACTGAGCCCTCG	
BG11-F	CCATGCAAATTGAGAAGAATGCC	Real-time PCR for *AhBG11*
BG11-R	CAAACCAATCTTGACTGTGG	
BG24-F	AGATCAGCTTTGTGGTTC	Real-time PCR for *AhBG24*
BG24-R	GTAATTTAGTTGCAGACGC	
CYP707A3-F	ATATGGAGAAAAGGGTGAC	Real-time PCR for *AhCYP707A3*
CYP707A3-R	CTATGTACACTTGAGATCCC	
UGT71K1-F	AAGAGATGGCTAGGAAGGC	Real-time PCR for *AhUGT71K1*
UGT71K1-R	TCTTTGTTCGTTTGATGGG	
UGT73B4-F	AAGAGCTGTTGAAGAAGGTGG	Real-time PCR for *AhUGT73B4*
UGT73B4-R	CTTCTTTGTATTGTTGGGC	
ABCG22-1-F	ATGAAGCTTCAATCCGGAGC	Real-time PCR for *AhABCG22-1*
ABCG22-1-R	CACATCAATGTAGTCCTC	
ABCG22-2-F	TTTCCTTGCGTTGGATG	Real-time PCR for *AhABCG22-2*
ABCG22-2-R	TCCCATCAGTGCTTGGCAC	

### Sequence analyses and alignments

The Gene Runner (Hastings Software, Inc., New York, USA) was used to perform the routine sequence analyses. Web-based analyses of cDNAs and deduced amino acid sequences were carried out using the Basic Local Alignment Search Tool (BLAST) program at the National Center for Biotechnology Information Services [44]. Multiple alignments of deduced amino acid sequences from target genes were performed by using the Clustal W program in the BioEdit software (Isis Pharmaceuticals, Inc., Carlsbad, USA). The full-length protein sequences were phylogenetically analyzed by using the MEGA 4 software with a bootstrapping set of 1000 replicates [45]. The subcellular localization of target proteins was predicted by using the iPSORT algorithm [46] at the website: http://ipsort.hgc.jp/ and the WoLF PSORT tool at the website: http://www.genscript.com/wolf-psort.html.

### Real-time quantitative RT-PCR performance

The isolated RNA by using the modified phenol chloroform method as previously described [33] was treated with RNase-free DNase I (TaKaRa, Dalian, China) at 37°C for 1 h to eliminate DNA contamination in real-time quantitative RT-PCR analysis. Reverse transcriptions (RT) were performed through the cDNA synthesis kit (TaKaRa, Dalian, China) according to the manufacturer’s and previously described protocols [18]. To investigate the expressions of target genes in peanut leaves in response to drought, the gene-specific primers were designed and listed in Table 1 to amplify the each corresponding cDNA for real-time quantitative PCR. As an internal control for normalization of target gene expression, the primers 18S-F (5’-ATT CCT AGT AAG CGC GAG TCA TCA G-3’) and 18S-R (5’-CAA TGA TCC TTC CGC AGG TTC AC-3’) specific to peanut 18S rRNA gene (GenBank accession no. AF156675) were used to amplify a fragment of 226 bp. Real-time quantitative PCRs were performed in the presence of Power SYBR green PCR Master Mix (Applied Biosystems, Guangzhou, China). Amplification was monitored in real-time with the MiniOpticon^TM^ Real-Time PCR System (Bio-Rad, Shanghai, China). The products of real-time quantitative PCR were confirmed by determining the melt curves for the products at the end of each run, by analysis of the products using gel electrophoresis, and by sequencing. The comparative cycle threshold (Ct) method was used to quantify the normalized gene expression biologically and technically with three replicates [47]. All RT-PCR data were expressed as the mean ± standard error. Statistical differences in target genes’ expression were assessed by one-way analysis of variance (ANOVA) followed by the least significant difference (LSD) and Student-Neumann-Keuls (SNK) post hoc comparison through SPSS 13.0 software (SPSS Inc., Chicago, IL, USA) with the threshold of significance defined as p<0.05.

### Measurement of endogenous ABA level

Endogenous ABA was isolated from the frozen leaf sample as described by Xiong et al [12]. Extraction in 80% (v/v) aqueous methanol, pre-purification through SepPak C18 cartridges (Waters, Milford, MA, USA), HPLC fractionation in a Kromasil C18 column (150×4.6 mm, 5 μm, Chenhang company, Shenzhen, China), and quantification of endogenous ABA were performed as reported previously [34,48]. The ABA level was determined triplicately with three replicates for each.

## Results and discussion

### Characterization of genes encoding enzymes involved in ABA production, catabolism and transport from peanut

From the constructed transcriptome of three-leaf-stage peanut leaves in response to drought [38], fourteen candidate genes involved in ABA production (*AhZEP*, *AhNCED1* and *AhNCED3*, *AhABA2*, *AhAAO1* and *AhAAO2*, *AhABA3*, *AhBG11* and *AhBG24*), catabolism (*AhCYP707A3*, *AhUGT71K1* and *AhUGT73B4*) and transport (*AhABCG22-1* and *AhABCG22-2*), were screened and identified homologously and phylogenetically. The characteristics of the full-length cDNAs of fourteen screened target genes obtained by RACE and the corresponding deduced proteins were shown in Table 2.

**Table 2:** Characteristics of full-length cDNAs of fourteen screened target genes and corresponding deduced proteins.

Gene	Accession no.	cDNA full-length (bp)	Open read frame (ORF) (bp)	5’ Untranslated region (UTR) (bp)	3’ UTR (bp)	Amino acid residues of deduced protein	Molecular weight of deduced protein (kDa)	Isoelectric point of deduced protein	Subcellular localization of deduced protein
*AhZEP*	MH037805	495	270	111	111	90	10.15	4.06	Chloroplast
*AhNCED1*	AJ574819	2486	1803	187	493	601	66.86	8.39	Chloroplast
*AhNCED3*	MH037806	2263	1917	181	162	639	70.88	6.91	Chloroplast
*AhABA2*	MH037807	1082	852	130	97	284	30.23	6.60	Cytosol
*AhAAO1*	EU183360	4585	3801	326	455	1267	138.04	6.38	Cytosol
*AhAAO2*	EU816196	4677	4080	327	267	1360	149.41	6.55	Cytosol
*AhABA3*	MH037808	3719	2448	112	1156	816	91.0	6.93	Cytosol
*AhBG11*	MH037809	1970	1539	228	200	513	58.84	5.68	Endoplasmic reticulum (ER)
*AhBG24*	MH037810	1763	1488	61	211	496	55.86	8.40	Vacuolar
*AhCYP707A3*	MH037811	1930	1392	163	372	464	53.15	9.29	ER
*AhUGT71K1*	MH037812	1628	1440	54	131	480	53.62	5.65	Cytosol
*AhUGT73B4*	MH037813	1607	1452	87	65	484	54.41	5.42	Cytosol
*AhABCG22-1*	MH037814	2572	2253	154	162	751	83.10	8.98	Plasma membrane
*AhABCG22-2*	MH037815	2429	2193	66	167	731	81.12	7.89	Plasma membrane

The main pathways of *de novo* ABA biosynthesis occur both in plastids and in the cytosol, starting from the precursor isopentenyl diphosphate (IPP), which is synthesized primarily in plastids from glyceraldehyde 3-phosphate and pyruvate, resulting in the successive production of the intermediates phytoene and lycopene [2,3]. Cyclization and hydroxylation of lycopene produce the oxygenated carotenoid zeaxanthin, which is then catalyzed by zeaxanthin epoxidase (ZEP) encoded by the *Arabidopsis AtABA1* locus to synthesize the violaxanthin [49]. In the present study, from our constructed drought-induced transcriptome of peanut leaves, one candidate *ZEP* was identified as *AhZEP*, encoding the enzyme AhZEP which shared 81%, 79%, 78%, 73% and 73% sequence identity with *Glycine soja* GsZEP (KHN42080), *Vigna radiata* VrZEP1 (XP_022631763), *Medicago truncatula* MtZEP (XP_013453497), *Medicago sativa* (AIP98334), and *Lupinus luteus* LlZEP (AHI87686), respectively. AhZEP protein was predicted by the iPSORT algorithm to have a chloroplast transit peptide MMPMMLSWVLGGNSSKLEGRPVCCRLSDKA at the N-terminus.

**Fig 1:**
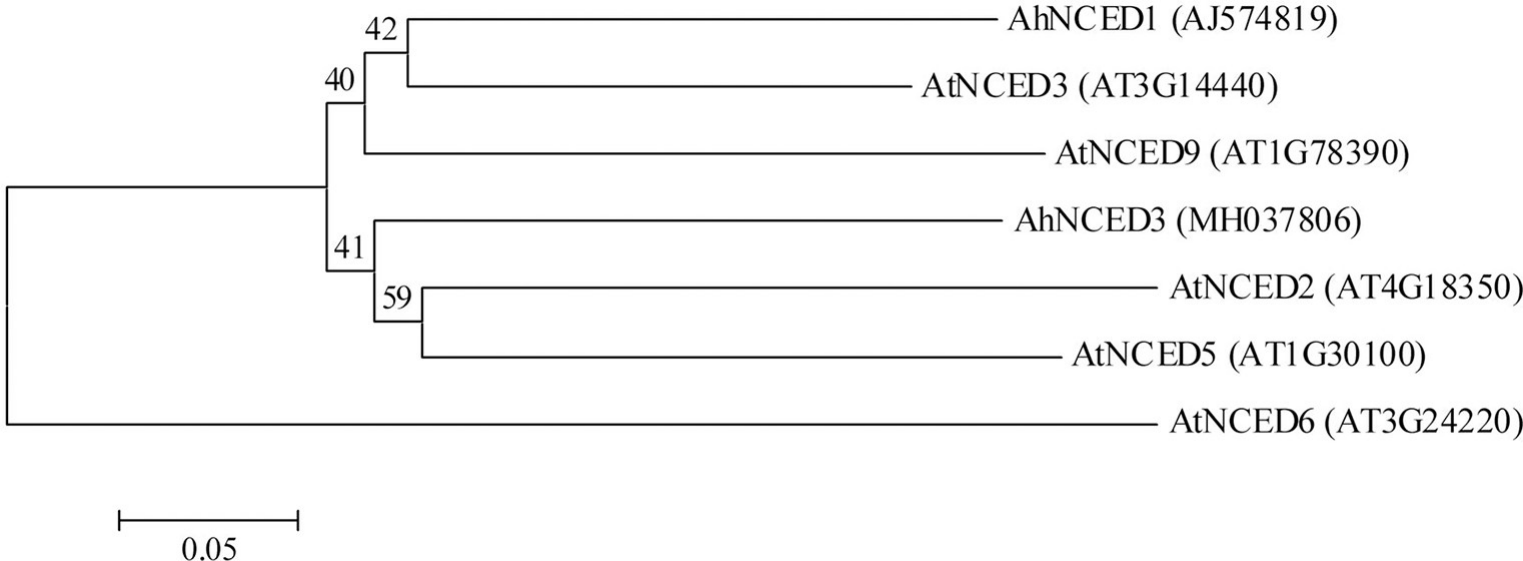
Phylogenetic analysis of amino acid sequences deduced from *AhNCED1*, *AhNCED3*, and five *Arabidopsis NCEDs* (*AtNCED2*, *3*, *5*, *6* and *9*). Multiple sequence alignment was performed using Clustal W and phylogenetic tree was constructed via the Neighbor-Joining method in MEGA 4 software. Bootstrap values from 1000 replicates for each branch were shown. GenBank accession numbers for each aligned NCED sequence were indicated in parentheses. The scale bar is 0.05.

In *Arabidopsis*, five AtNCEDs (AtNCED2, 3, 5, 6 and 9) were characterized to cleave the substrates violaxanthin and neoxanthin to a C_15_ product, xanthoxin (the first cytoplasmic precursor in ABA biosynthetic pathway) [50]. Here two candidate *NCED* genes, *AhNCED1* (our previous work [33,34]) and *AhNCED3* were characterized from the constructed drought-induced transcriptome of peanut leaves. Multiple alignments showed that the deduced amino acids from *AhNCED1* and *AhNCED3* shared 59.2% sequence identity with each other. AhNCED3 protein shared 60.2%, 62.2%, 61.9%, 47.9% and 54.7% sequence identity with *Arabidopsis* AtNCED2, 3, 5, 6 and 9, respectively. A putative 30-amino-acid chloroplast transit peptide MIMAPSSIALNSASSSTWAKKPHQLSRPFS predicted by the iPSORT algorithm is located at the N-terminus of AhNCED3 protein, structurally similar with reported NCED proteins [3,33,50,51]. Phylogenetic analysis of AhNCED1, AhNCED3 and five *Arabidopsis* NCEDs showed that AhNCED1 and AtNCED3 were clustered into one group (Fig 1), both of them playing a vital role in stress-induced ABA biosynthesis in leaves [34,50]. AhNCED3 was clustered with AtNCED2 and AtNCED5 (Fig 1), which accounted for the main *NCED* transcripts in flowers [50].

**Fig 2:**
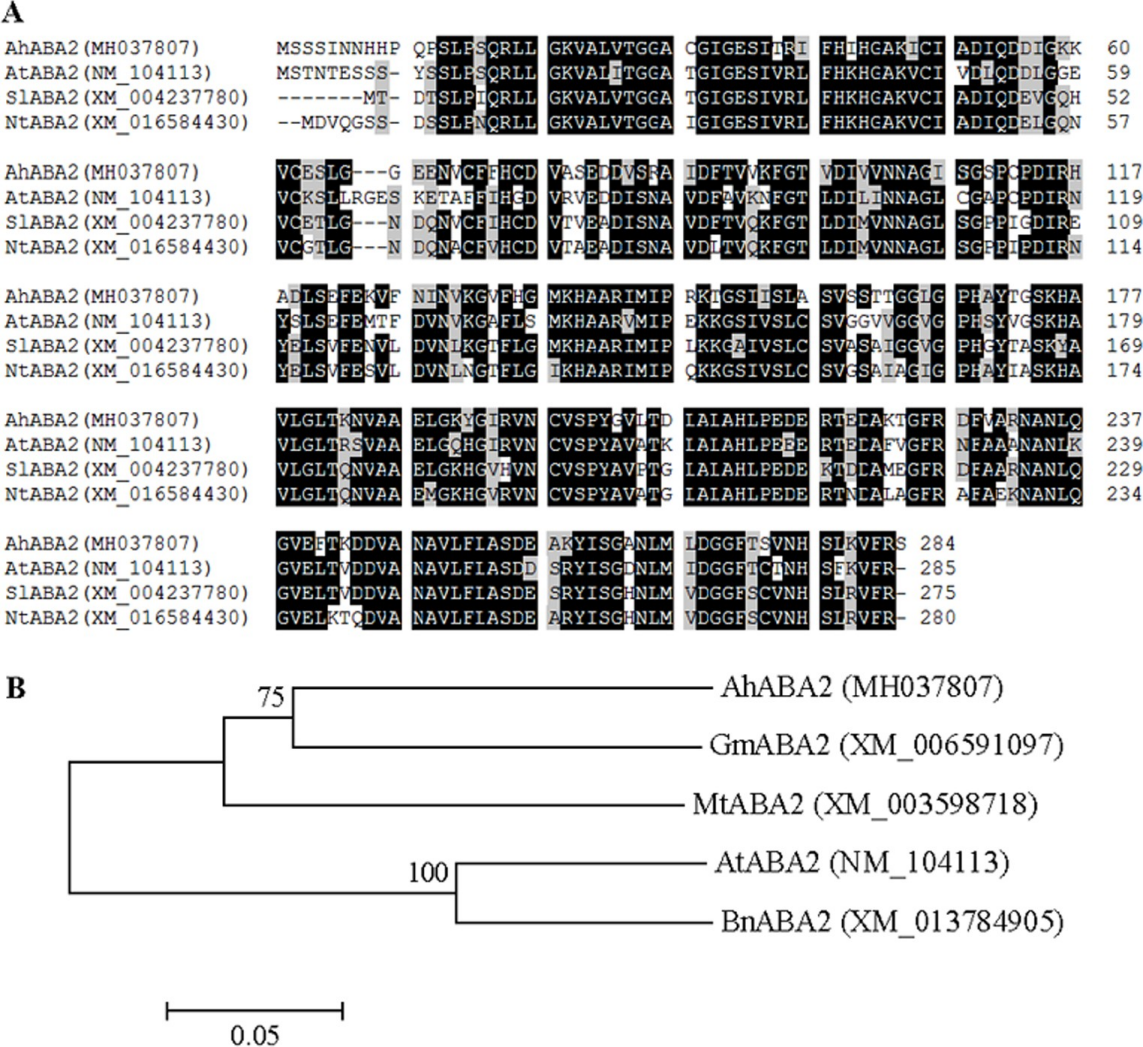
Sequence analyses of ABA2 proteins from peanut, *Arabidopsis*, tomato, tobacco, soybean, alfalfa, and winter rape. **(A)** Alignment of deduced amino acid sequences from peanut *AhABA2*, *Arabidopsis AtABA2*, tomato *SlABA2*, and tobacco *NtABA*2. Identical and similar amino acid residues were shaded in black and gray, respectively. Dotted lines indicated gaps that were introduced to maximize the alignment. Amino acids were numbered from the initial methionine. GenBank accession numbers for each aligned ABA2 homolog were indicated in parentheses. **(B)** Phylogenetic analysis of amino acid sequences of AhABA2, AtABA2, soybean GmABA2, alfalfa MtABA2, and winter rape BnABA2. Multiple sequence alignment was performed using Clustal W and phylogenetic tree was constructed via the Neighbor-Joining method in MEGA 4 software. Bootstrap values from 1000 replicates for each branch were shown. GenBank accession numbers for each analyzed ABA2 were indicated in parentheses. The scale bar is 0.05.

The conversion of xanthoxin into abscisic aldehyde is catalyzed by AtABA2 in *Arabidopsis*, which belongs to the short-chain dehydrogenases/reductases (SDR) family [7,8]. A severe ABA deficiency resulting from loss of function of *AtABA2* suggests that AtABA2 protein appears to be encoded by a single gene in *Arabidopsis* genome [8]. In the present study, *AhABA2* was characterized to encode AtABA2 homolog in peanut. Multiple alignments showed that AhABA2 protein shared 67.2%, 70% and 67.9 sequence identity with AtABA2, tomato SlABA2 and tobacco NtABA2, respectively (Fig 2A). The domain (residues 3 to 285 in AtABA2) with xanthoxin dehydrogenase activity was highly conserved in all aligned ABA2 proteins (Fig 2A). AhABA2 was phylogenetically closer to soybean GmABA2 in the leguminous cluster (Fig 2B).

**Fig 3:**
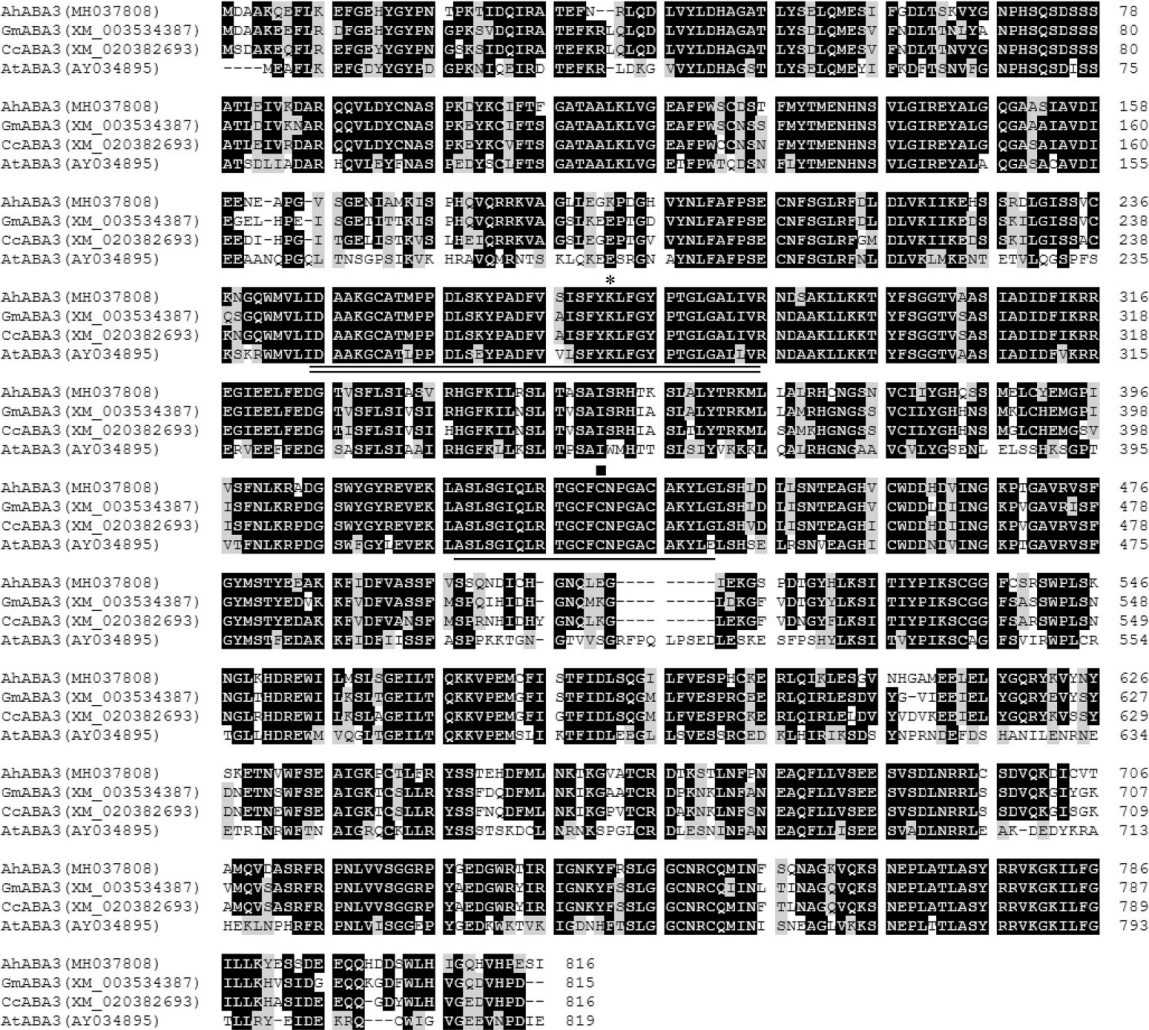
Alignment of deduced amino acid sequences from peanut *AhABA3*, soybean *GmABA3*, *Cajanus cajan CcABA3* and *Arabidopsis AtABA3*. Identical and similar amino acid residues were shaded in black and gray, respectively. Dotted lines indicated gaps that were introduced to maximize the alignment. The conserved cysteine motif was underlined and the putative PLP binding motif was double underlined. The conserved critical lysine residue in the PLP domain was indicated with an upper asterisk, and the conserved cysteine residue was indicated with an upper square. Amino acids were numbered from the initial methionine. GenBank accession numbers for each aligned ABA3 homolog were indicated in parentheses.

The oxidation of abscisic aldehyde to ABA, which is catalyzed by abscisic aldehyde oxidase, is the final step in ABA biosynthetic pathway. Among four abscisic aldehyde oxidases (AtAAO1 to 4) in *Arabidopsis*, AtAAO3 was reported to actively utilize abscisic aldehyde as a substrate, most probably the only one AAO involved in ABA biosynthesis [11]. Here our previously characterized two peanut *AAO* genes, *AhAAO1* [52] and *AhAAO2* [53], were also screened from the constructed drought-induced transcriptome of peanut leaves. AhAAO1 protein was predicted to localize in the cytosol by the WoLF PSORT tool, and AhAAO2 was predicted by the iPSORT algorithm as not having any of signal, mitochondrial targeting, or chloroplast transit peptides. The aldehyde oxidase requires a molybdenum cofactor (MoCo) for its catalytic activity. To date, *AtABA3* (a single-copy gene in the genome) was the only reported *ABA3* gene encoding *Arabidopsis* sulfurase that produces a functional cofactor [12]. In this study, an *AtABA3* homolog gene *AhABA3* was characterized from the drought-induced transcriptome of peanut leaves. Multiple alignments showed that AhABA3 protein shared 82.1%, 80.9% and 61.3% sequence identity with soybean GmABA3, *Cajanus cajan* CcABA3 and AtABA3, respectively (Fig 3). The putative pyridoxal phosphate (PLP) binding motif and the conserved cysteine motif identified by Xiong et al [12] both exist in AhABA3 protein sequence (Fig 3).

**Fig 4:**
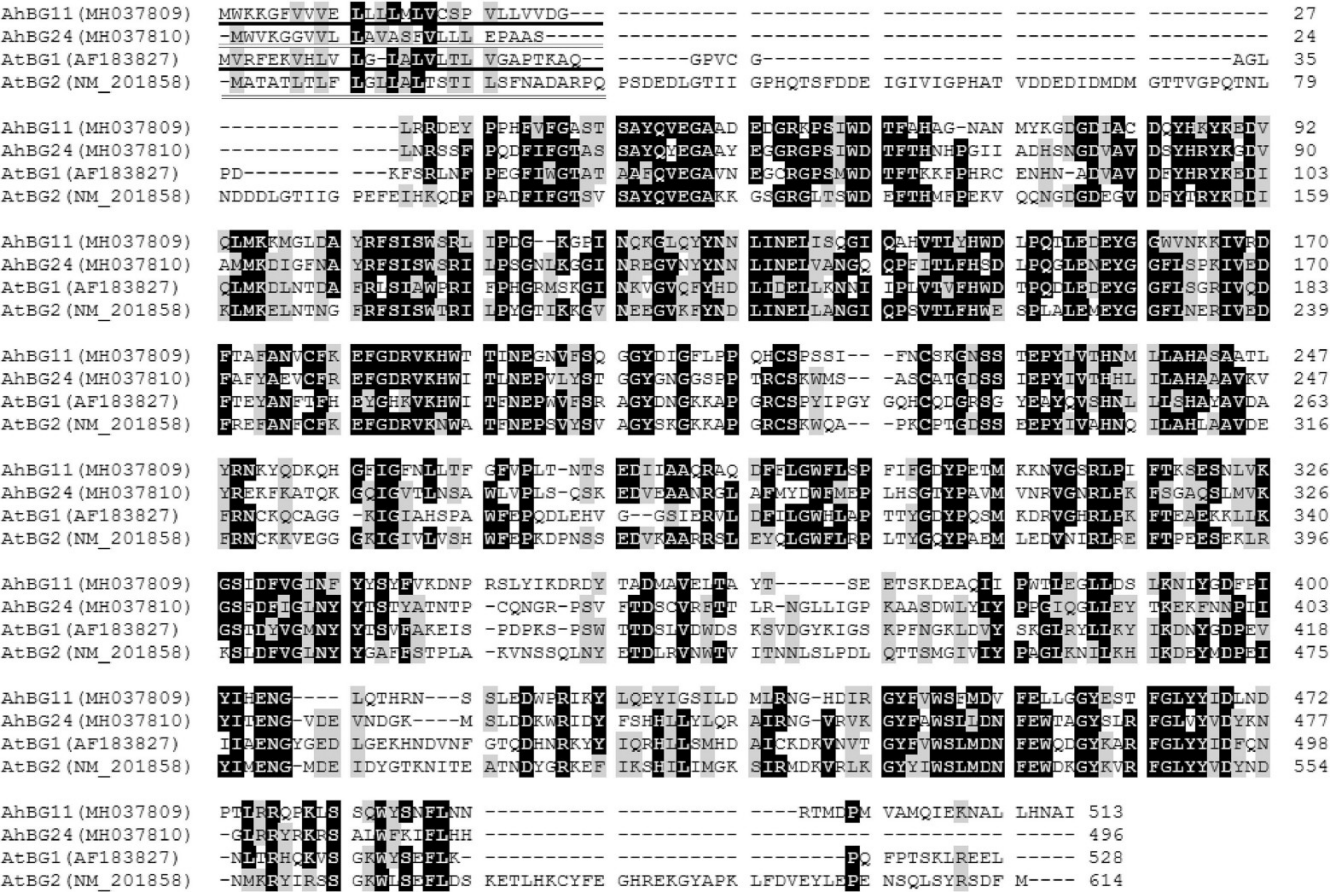
Alignment of deduced amino acid sequences from peanut *AhBG11*, *AhBG24*, and *Arabidopsis AtBG1*, *AtBG2*. Identical and similar amino acid residues were shaded in black and gray, respectively. Dotted lines indicated gaps that were introduced to maximize the alignment. Putative ER-localization signal peptide was underlined in AtBG1 and AhBG11 [13]; and putative vacuolar-targeting motif was double underlined in AtBG2 and AhBG24 [14]. Amino acids were numbered from the initial methionine. GenBank accession numbers for each aligned BG homolog were indicated in parentheses.

The hydrolysis of ABA-GE catalyzed by β-glucosidase (BG) is an alternative pathway to produce ABA. The β-glucosidase homologs, *Arabidopsis* AtBG1 and AtBG2, localize to the ER and vacuole, respectively [13,14]. AtBG2 belongs to the same subfamily as AtBG1 that consists of 16 members in the large number of β-glucosidases found in *Arabidopsis* [13,54], which can be divided into two groups: AtBG1 belongs to the group of seven members with an ER retrieval signal, and AtBG2 belongs to the other group of nine members without the ER retrieval signal [14]. In the present study, two *BG* homologs, *AhBG11* and *AhBG24*, were characterized from our constructed drought-induced transcriptome of peanut leaves. AhBG11 protein shared 41.6%, 37.7% and 32.5% sequence identity with AhBG24, AtBG1 and AtBG2, respectively; and AhBG24 shared 40.2% and 37.2% sequence identity with AtBG1 and AtBG2, respectively (Fig 4). AhBG11 and AhBG24 were predicted by the WoLF PSORT tool to localize to the ER and vacuole, respectively (Table 2; Fig 4), suggesting that AhBG11 and AhBG24 might belong to the group with AtBG1 and the other group with AtBG2, respectively.

**Fig 5:**
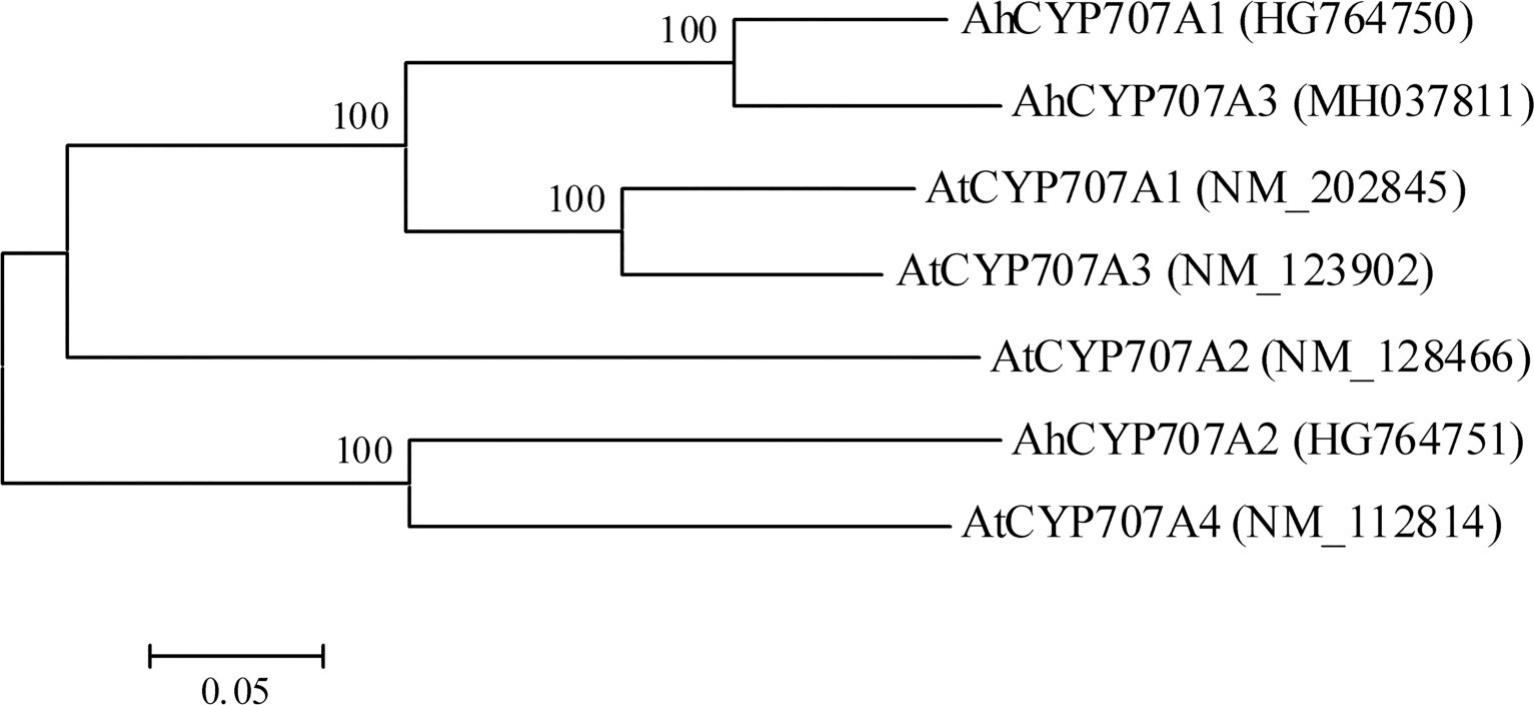
Phylogenetic analysis of amino acid sequences deduced from three peanut (*AhCYP707A1*, *2* and *3*) and four *Arabidopsis* (*AtCYP707A1*, *2*, *3* and *4*) *CYP707A* genes. Multiple sequence alignment was performed using Clustal W and phylogenetic tree was constructed via the Neighbor-Joining method in MEGA 4 software. Bootstrap values from 1000 replicates for each branch were shown. GenBank accession numbers for each aligned CYP707A sequence were indicated in parentheses. The scale bar is 0.05.

The catabolic process of ABA mainly involves two pathways, hydroxylation and glucose conjugation. The 8′-hydroxylation of ABA is the predominant enzymatic reaction, which is mediated by the protein encoded by AtCYP707A gene family (*AtCYP707A1*, *2*, *3* and *4*) in *Arabidopsis* [15]. In this study, from our transcriptome, another peanut *CYP707A* gene, *AhCYP707A3* was identified, and AhCYP707A3 protein shared 84.4%, 50.9%, 65%, 54%, 68.2% and 53% sequence identity with AhCYP707A1, 2 (our previously characterized two peanut CYP707As [18]), and AtCYP707A1, 2, 3 and 4, respectively. Like AhCYP707A1 and 2, AhCYP707A3 contains the highly conserved cysteine motif (PFGNGTHSCPG), which was reported to be essential for the hydroxylation [55]. Three peanut CYP707A proteins (AhCYP707A1, 2 and 3) were all predicted as having a signal peptide by the iPSORT algorithm, consistent with the report of ER-membrane localized ABA catabolism catalyzed by CYP707As [20]. In the phylogenetic tree (Fig 5), AhCYP707A1, 3 and AtCYP707A1, 3 proteins were clustered into one group, and AhCYP707A3 was relatively closer to AhCYP707A1, consistent with the above result of sequence identity analysis.

**Fig 6:**
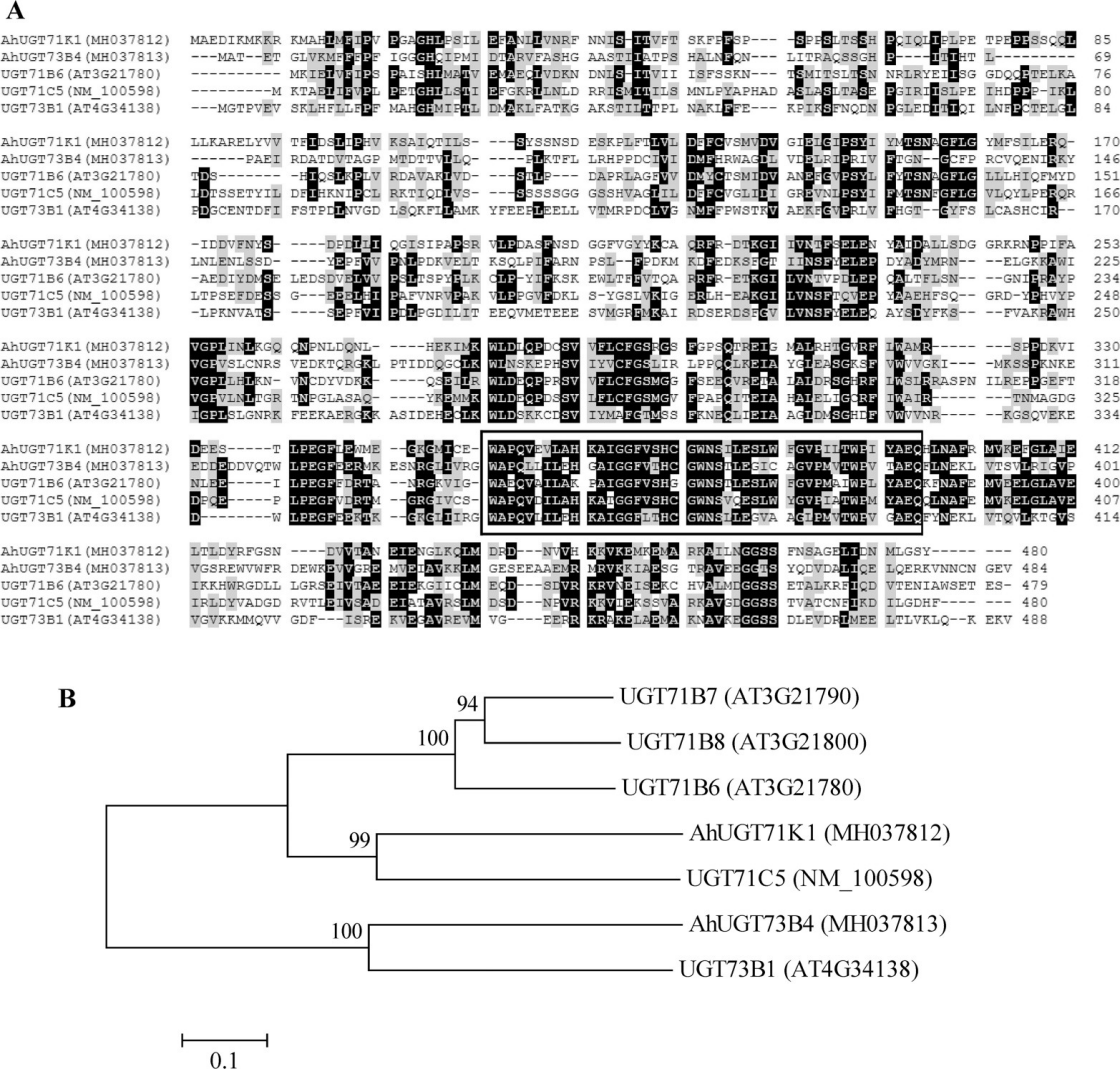
Sequence analyses of UGT proteins from peanut and *Arabidopsis*. **(A)** Alignment of deduced amino acid sequences from peanut *AhUGT71K1*, *AhUGT73B4* and *Arabidopsis UGT71B6*, *UGT71C5*, *UGT73B1*. Identical and similar amino acid residues were shaded in black and gray, respectively. Dotted lines indicated gaps that were introduced to maximize the alignment. The highly conserved motif UDPGT in all UGTs was boxed. Amino acids were numbered from the initial methionine. GenBank accession numbers for each aligned UGT homolog were indicated in parentheses. **(B)** Phylogenetic analysis of amino acid sequences of peanut AhUGT71K1, AhUGT73B4 and *Arabidopsis* UGT71B6, UGT71B7, UGT71B8, UGT71C5, UGT73B1. Multiple sequence alignment was performed using Clustal W and phylogenetic tree was constructed via the Neighbor-Joining method in MEGA 4 software. Bootstrap values from 1000 replicates for each branch were shown. GenBank accession numbers for each analyzed UGT were indicated in parentheses. The scale bar is 0.1.

The main conjugation pathway for ABA is glucosylation catalyzed by ABA UDP-glucosyltransferases (UGTs), which produces ABA-GE, a storage form and an inactive end product of ABA metabolism [56,57]. Previously reported UGTs, UGT71B6, UGT71B7 and UGT71B8, UGT73B1 and UGT73B3, UGT75B1 and UGT75B2, UGT84B1 and UGT84B2, which displayed *in vitro* the activity to glucosylate ABA, belong to the UGT subfamilies of the family 1 in *Arabidopsis* [58]. In the present study, two unique ABA *UGT* genes, *AhUGT71K1* and *AhUGT73B4*, were identified from the constructed drought-induced transcriptome of peanut leaves. Multiple alignments showed that AhUGT71K1 protein shared the highest sequence identity with *Arabidopsis* UGT71C5, which was very recently confirmed *in vitro* and *in vivo* to play a major role in ABA glucosylation for ABA homeostasis [58]. AhUGT73B4 shared the highest sequence identity with *Arabidopsis* UGT73B1, which displayed ABA glucosylation activity *in vitro* [58]. A motif, named as UDPGT [59], involved in binding to the donor sugar was highly conserved in the C-terminal sequences of all analyzed UGT proteins (Fig 6A). AhUGT71K1 and AhUGT73B4 were both predicted by the WoLF PSORT tool to localize in the cytosol, similar to the cytosolic localization of UGT71B6, UGT71B7, UGT71B8 and UGT71C5 [17,58]. Consistent with the result of sequence alignment, AhUGT71K1 and AhUGT73B4 were phylogenetically closer to UGT71C5 and UGT73B1, respectively (Fig 6B).

**Fig 7:**
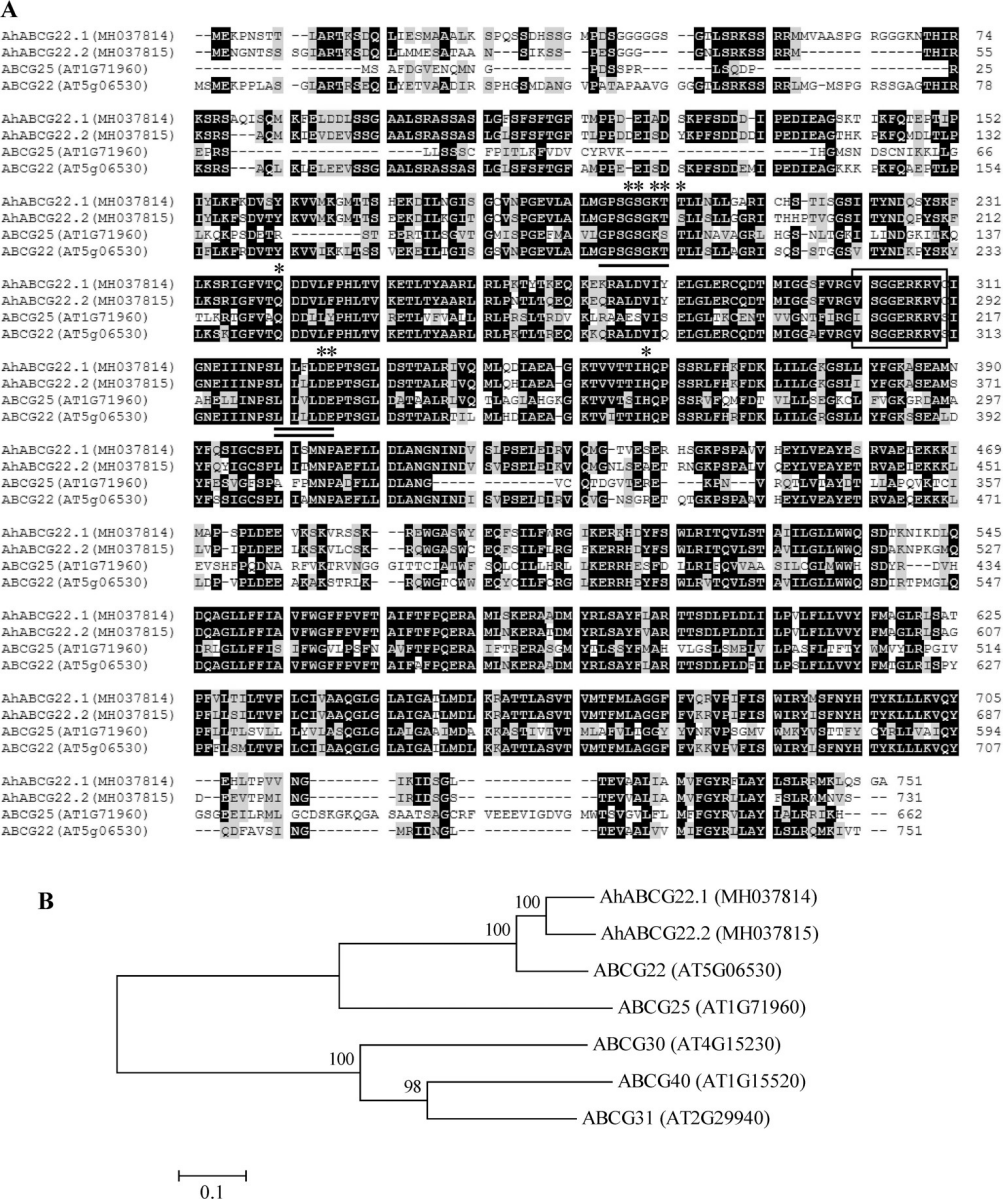
Sequence analyses of ABCG proteins from peanut and *Arabidopsis*. **(A)** Alignment of deduced amino acid sequences from peanut *AhABCG22*.*1*, *AhABCG22*.*2* and *Arabidopsis ABCG25*, *ABCG22*. Identical and similar amino acid residues were shaded in black and gray, respectively. Dotted lines indicated gaps that were introduced to maximize the alignment. The highly conserved features of ATP-binding site, ABC transporter signature motif, Walker A/P-loop and Walker B in all ABCGs were respectively indicated with asterisks, a box, an underline and a double-underline. Amino acids were numbered from the initial methionine. GenBank accession numbers for each aligned ABCG homolog were indicated in parentheses. **(B)** Phylogenetic analysis of amino acid sequences of peanut AhABCG22.1, AhABCG22.2 and *Arabidopsis* ABCG25, ABCG40, ABCG22, ABCG30, ABCG31. Multiple sequence alignment was performed using Clustal W and phylogenetic tree was constructed via the Neighbor-Joining method in MEGA 4 software. Bootstrap values from 1000 replicates for each branch were shown. GenBank accession numbers for each analyzed ABCG were indicated in parentheses. The scale bar is 0.1.

The translocation of ABA between cells, tissues and organs also plays important roles in whole plant physiological response to stress conditions. ABA can diffuse passively across biological membranes when it is protonated [21,60], and can also be transported across plasma membranes by ABCG transporters [61,62]. To date, at least eight different ABA transporters have been identified by genetic and functional screening [22–29]. In the present study, two *ABCG* gene homologs, *AhABCG22*.*1* and *AhABCG22*.*2* were screened and characterized from our constructed drought-induced transcriptome of peanut leaves. Multiple alignments showed that AhABCG22.1 and AhABCG22.2 proteins shared 81% mutual sequence identity; AhABCG22.1 shared 75.6% and 36.7% sequence identity with *Arabidopsis* ABCG22 and ABCG25, respectively; AhABCG22.2 shared 75.3% and 37.8% sequence identity with *Arabidopsis* ABCG22 and ABCG25, respectively. The characterized domains ABC transporter G-25 (residues 111–746 and 123–726 respectively in AhABCG22.1 and AhABCG22.2) and ABC2_membrane (residues 501–703 and 483–685 respectively in AhABCG22.1 and AhABCG22.2) were highly conserved; the conserved features of ATP-binding site, ABC transporter signature motif, Walker A/P-loop and Walker B were also found in both AhABCG22.1 and AhABCG22.2 (Fig 7A). AhABCG22.1 and AhABCG22.2 were both predicted subcellularly as integral plasma membrane proteins. Phylogenetic tree of AhABCG22.1 and AhABCG22.2, and five *Arabidopsis* ABCGs (ABCG25, ABCG40, ABCG22, ABCG30 and ABCG31) demonstrated that AhABCG22.1 and AhABCG22.2 were clustered with ABCG22, and that all three were relatively closer to ABCG25 (Fig 7B).

### Expression pattern of genes involved in ABA production, catabolism and transport in peanut leaves in response to drought stress

**Fig 8:**
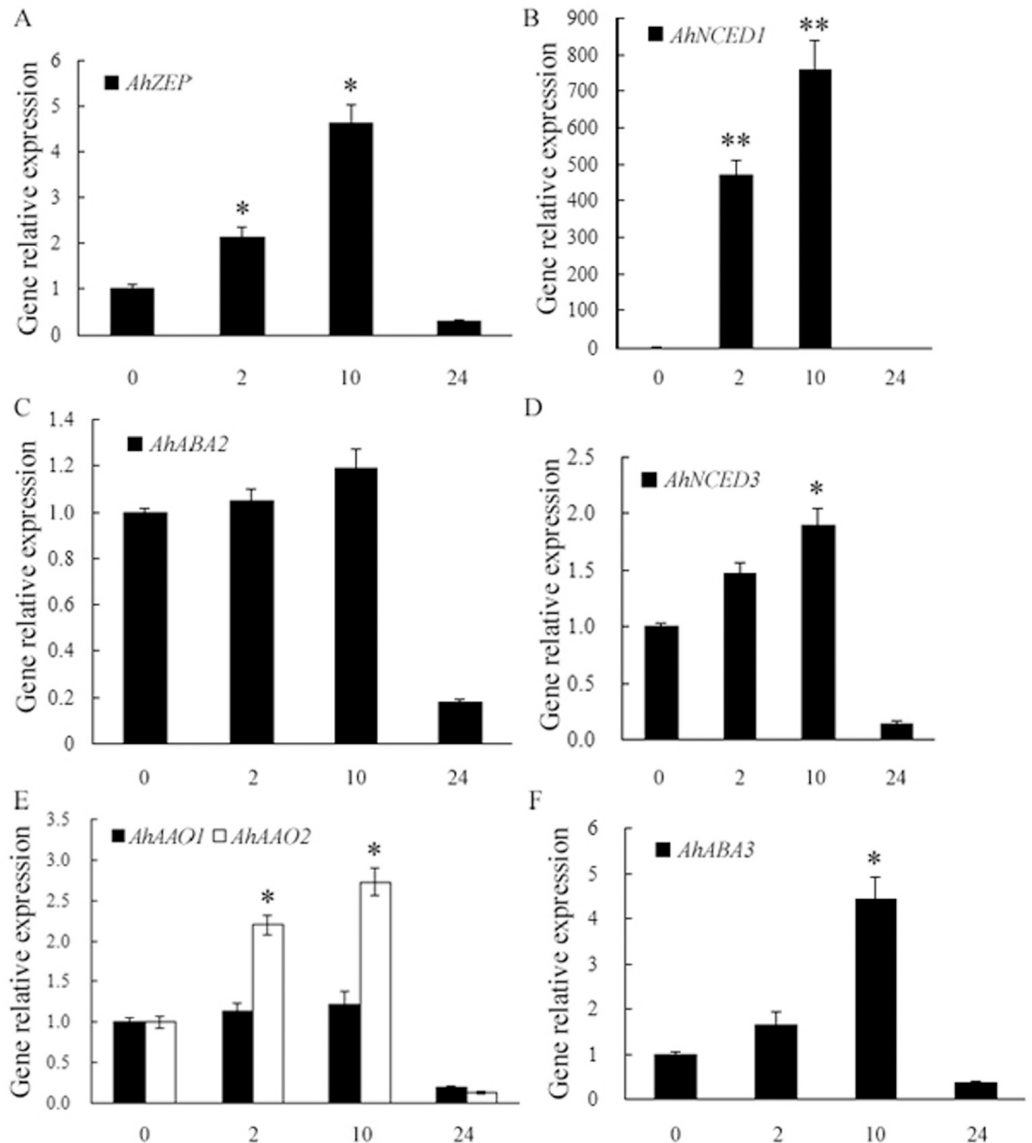
**Expressions of ABA biosynthetic genes, including *AhZEP* (A), *AhNCED1* (B) and *AhNCED3* (D), *AhABA2* (C), *AhAAO1* and *AhAAO2* (E), and *AhABA3* (F) in peanut leaves in response to drought stress.** Peanut seedlings of twelve days old were hydroponically grown in the solution containing 20% PEG6000 or deionized water as a control for indicated time (The expressions of genes in peanut leaves during control conditions showed no obvious difference with that in 0 h stressed sample and were not presented). Total RNA was prepared respectively from leaves of control or stressed plants. Gene expressions detected by real-time quantitative RT-PCR were shown relative to the expression of peanut *18S rRNA* gene in each sample. All data are presented as mean ± standard errors (SE) of three replicates. The asterisk above each bar indicates a significant difference between stressed and controlled samples at *P* < 0.05 (*) or *P* < 0.01 (**).

It has been reported that, with the exception of *AtABA2*, the expressions of most of the genes involved in *de novo* biosynthesis of ABA are up-regulated by drought stress [8–12,49,63]. In contrast, *AtABA2* is expressed constitutively at a relatively low level and is not induced by dehydration stress [7,8]. In the present study, real-time RT-PCR was performed to detect the expressions of the above characterized genes involved in ABA biosynthetic pathway in peanut leaves in response to drought stress. The results showed that gene expressions of *AhZEP*, *AhNCED1*, *AhAAO2* and *AhABA3* were significantly up-regulated in response to drought stress (Fig 8). Particularly, the transcript level of *AhNCED1* gene was strongly increased by drought stress (756 times higher than that in the control at 10 h of the stress) (Fig 8B), consistent with our previous reports [18,34]. The expression of *AhNCED3* (Fig 8D) was also induced by drought (0.9 times higher than that in the control at 10 h of the stress), but the induction was much slighter than that of *AhNCED1* (Fig 8B, D). However, the expressions of *AhABA2* (Fig 8C) and *AhAAO1* (Fig 8E) were not affected significantly by the stress, which were consistent with the previous reports of *AtABA2* [7,8] and *AhAAO1* [52].

**Fig 9:**
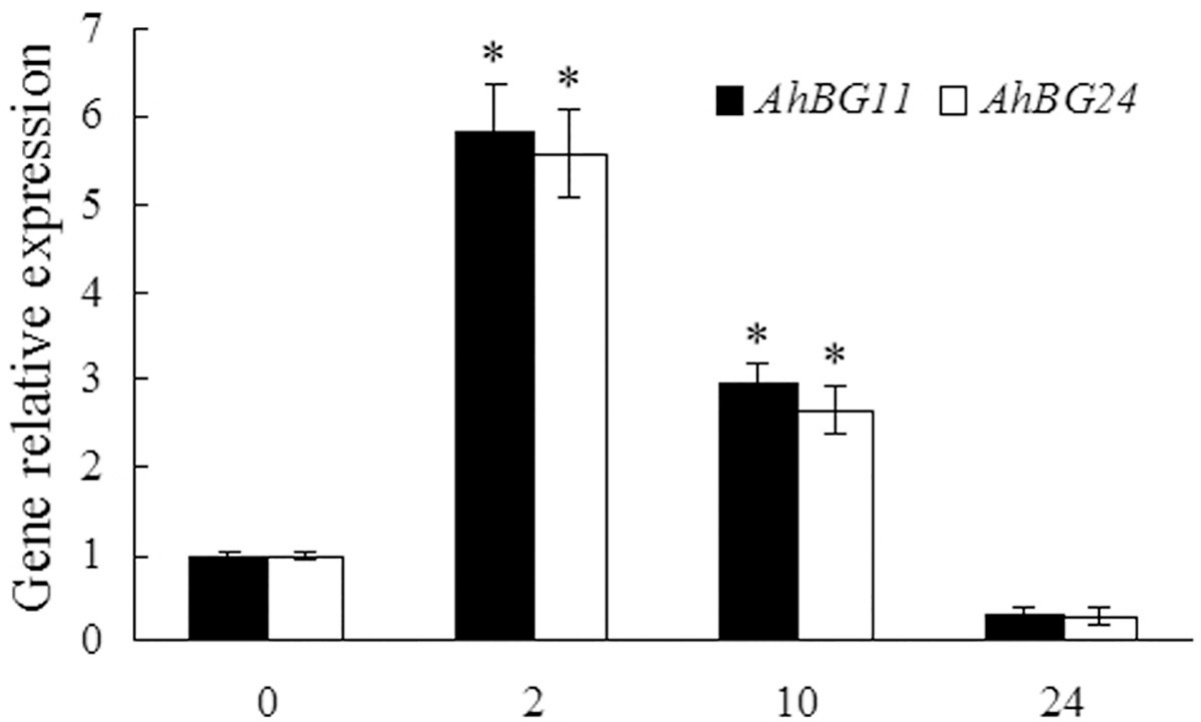
β-glucosidase coding genes, including *AhBG11* and *AhBG24* in peanut leaves rapidly and highly respond to drought stress. Peanut seedlings of twelve days old were hydroponically grown in the solution containing 20% PEG6000 or deionized water as a control for indicated time (The expressions of genes in peanut leaves during control conditions showed no obvious difference with that in 0 h stressed sample and were not presented). Total RNA was prepared respectively from leaves of control or stressed plants. Real-time RT-PCR analysis was performed as described in Fig 8. All data are presented as mean ± standard errors (SE) of three replicates. The asterisk above each bar indicates a significant difference between stressed and controlled samples at *P* < 0.05 (*).

Compared with the lengthy *de novo* biosynthetic pathway [3,56,64], the one-step hydrolysis of ABA-GE to ABA catalyzed by BG is a fast process, which is optimal to meet the rapid increase in ABA level in response to stresses. *Arabidopsis AtBG1* and *AtBG2* were both reported to be induced by dehydration stress [13,14]. Loss of *AtBG1* [13] or *AtBG2* [14] in *Arabidopsis* caused lower ABA levels and reduced abiotic stress tolerance, whereas overexpression of *AtBG1* [13] or *AtBG2* [14] resulted in higher ABA accumulation and enhanced tolerance to abiotic stress. In this study, the expressions of *AhBG11* and *AhBG24* genes in peanut leaves in response to drought stress were determined by real-time RT-PCR performance. As shown in Fig 9, the transcript levels of *AhBG11* and *AhBG24* were rapidly and significantly up-regulated by 2-h (4.83- and 4.58-fold increase, respectively) or 10-h (1.97- and 1.65-fold increase, respectively) drought stress.

**Fig 10:**
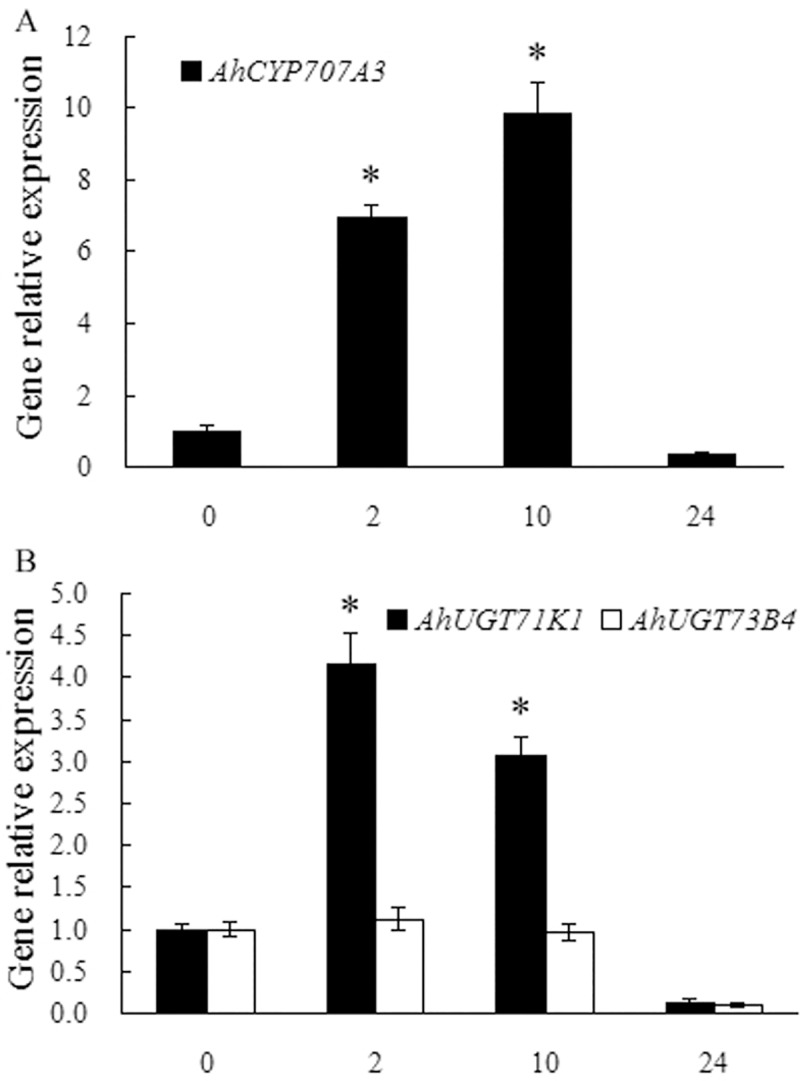
Expressions of ABA catabolic genes, including *AhCYP707A3*, *AhUGT71K1* and *AhUGT73B4* in peanut leaves in response to drought stress. Peanut seedlings of twelve days old were hydroponically grown in the solution containing 20% PEG6000 or deionized water as a control for indicated time (The expressions of genes in peanut leaves during control conditions showed no obvious difference with that in 0 h stressed sample and were not presented). Total RNA was prepared respectively from leaves of control or stressed plants. Real-time RT-PCR analysis was performed as described in Fig 8. All data are presented as mean ± standard errors (SE) of three replicates. The asterisk above each bar indicates a significant difference between stressed and controlled samples at *P* < 0.05 (*).

ABA catabolism is mediated through hydroxylation and glucose conjugation, and also plays important roles in regulating cellular ABA levels. The transcript levels of all four *Arabidopsis CYP707A* genes increased in response to mannitol or drought stress [15]. The *CYP707A5* mRNA level in rice leaves sharply responded to mannitol [65]. We previously demonstrated that the transcript levels of peanut *CYP707A1* and *2* genes increased in response to PEG6000- or NaCl-induced osmotic stress [18]. Here another peanut *CYP707A* gene, *AhCYP707A3* was shown to be significantly induced in leaves in response to drought stress, with a 5.93- or an 8.85-fold increase in the transcript respectively at 2 or 10 h of the stress (Fig 10A). The conjugation of ABA with glucose is catalyzed by UGT to produce ABA-GE [16,17]. In *Arabidopsis*, *UGT71B6* gene and its two homologs, *UGT71B7* and *UGT71B8* were all reported to be rapidly induced by osmotic stress [17]. Liu et al [58] showed that mutation of *UGT71C5* and down-expression of *UGT71C5* in *Arabidopsis* caused delayed seed germination and enhanced drought tolerance; and that overexpression of *UGT71C5* accelerated seed germination and reduced drought tolerance. In the present study, the expression of *AhUGT71K1*, highly phylogenetically similar to *UGT71B6* (Fig 6B), was rapidly and significantly up-regulated in peanut leaves in response to drought stress, with a 3.16- or 2.07-fold increase in the transcript respectively at 2 or 10 h of the stress (Fig 10B). Whereas, the transcript level of *AhUGT73B4* in peanut leaves did not respond to drought stress markedly (Fig 10B).

**Fig 11:**
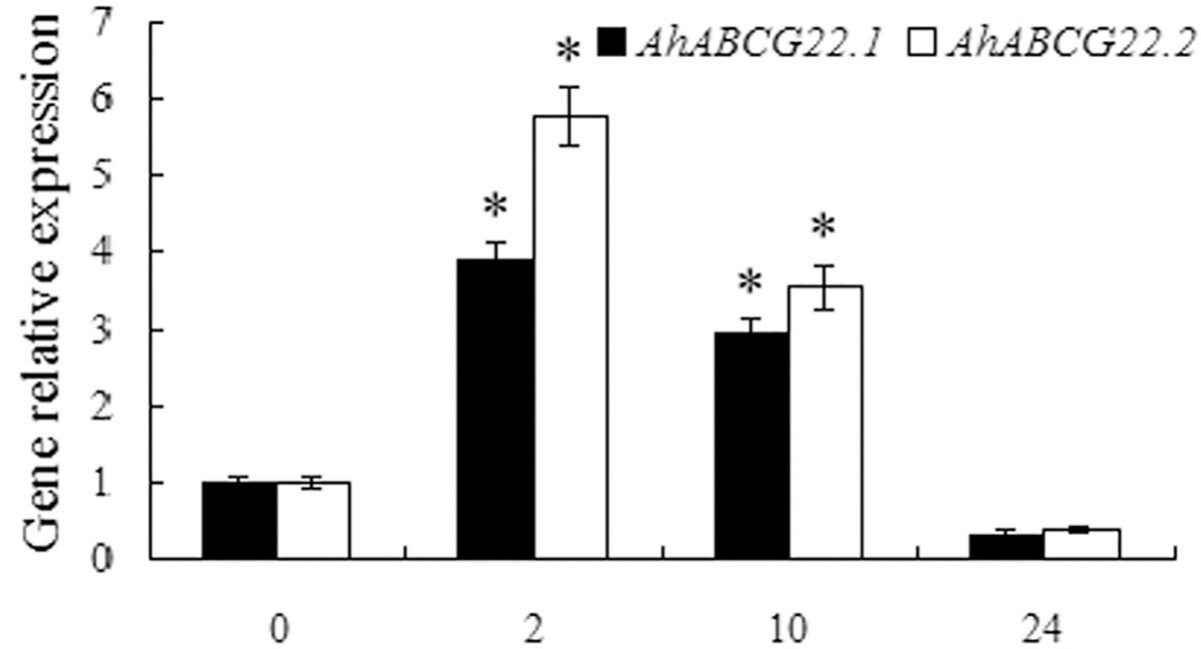
Drought stress significantly induces the expression of ABA importer genes, *AhABCG22*.*1* and *AhABCG22*.*2* in peanut leaves. Peanut seedlings of twelve days old were hydroponically grown in the solution containing 20% PEG6000 or deionized water as a control for indicated time (The expressions of genes in peanut leaves during control conditions showed no obvious difference with that in 0 h stressed sample and were not presented). Total RNA was prepared respectively from leaves of control or stressed plants. Real-time RT-PCR analysis was performed as described in Fig 8. All data are presented as mean ± standard errors (SE) of three replicates. The asterisk above each bar indicates a significant difference between stressed and controlled samples at *P* < 0.05 (*).

*Arabidopsis* ABCG25 and ABCG40 were shown to be responsible for ABA transport and response, which function as an ABA exporter and importer, respectively [22,23]. Recently, the removal of PM-localized ABCG25 via activation of endocytosis and transport to vacuole was confirmed to be another mechanism by which plant cells increase cellular ABA levels in response to abiotic stresses, in addition to the activation of ABA biosynthetic genes [66]. Kuromori et al [24] showed that *Arabidopsis* ABCG22 is required for stomatal regulation and involved in ABA influx. In this study, the expressions of two closely related *ABCG22* genes in peanut leaves, *AhABCG22*.*1* and *AhABCG22*.*2*, were significantly up-regulated by 2-h (2.89- and 4.77-fold increase, respectively) or 10-h (1.93- and 2.54-fold increase, respectively) drought stress (Fig 11), respectively. Under abiotic stress conditions, plant cells need to increase the cellular ABA levels to trigger ABA-mediated signaling in order to respond to the stresses [49,67], therefore the expression levels of genes involved in ABA production pathways are up-regulated to increase the cellular ABA levels [8–12,49,63] (Figs 8 and 9). At this condition, high levels of *AhABCG22* transcripts would contribute to the rapid increase of cellular ABA levels (Fig 11).

### Genes involved in ABA production, catabolism and transport jointly regulate ABA homeostasis in peanut leaves in response to drought

ABA production, catabolism, and transport all affect ABA homeostasis in plant cells [2]. Two production pathways, *de novo* biosynthesis and hydrolysis of glucose-conjugated ABA, increase the cellular ABA levels [3,13,14,56,64]. ABA catabolism via hydroxylation or conjugation decreases the cellular ABA levels [62]. Although extensive work has been performed on the hydroxylation pathway, little is known about the conjugation pathway. In particular, the contribution of conjugation pathway in ABA homeostasis regulation has been less clear. Recently, the determination of ABA content in *Arabidopsis* showed that mutation in *UGT71C5* and down-expression of *UGT71C5* resulted in increased level of ABA, whereas overexpression of *UGT71C5* resulted in reduced level of ABA [58]. The transport of ABA through ABCGs across the plasma membrane is another important pathway to regulate cellular ABA homeostasis [22–24,62]. Consistent with this proposed activity, the ABA exporter *atabcg25* mutants displayed ABA hypersensitive phenotypes at different developmental stages [22]. In contrast, AtABCG40/AtPDR12 is responsible for ABA uptake, which is consistent with the phenotype of *atabcg40*/*atpdr12* that showed a defect in stomatal closure and enhanced water loss [23].

**Fig 12:**
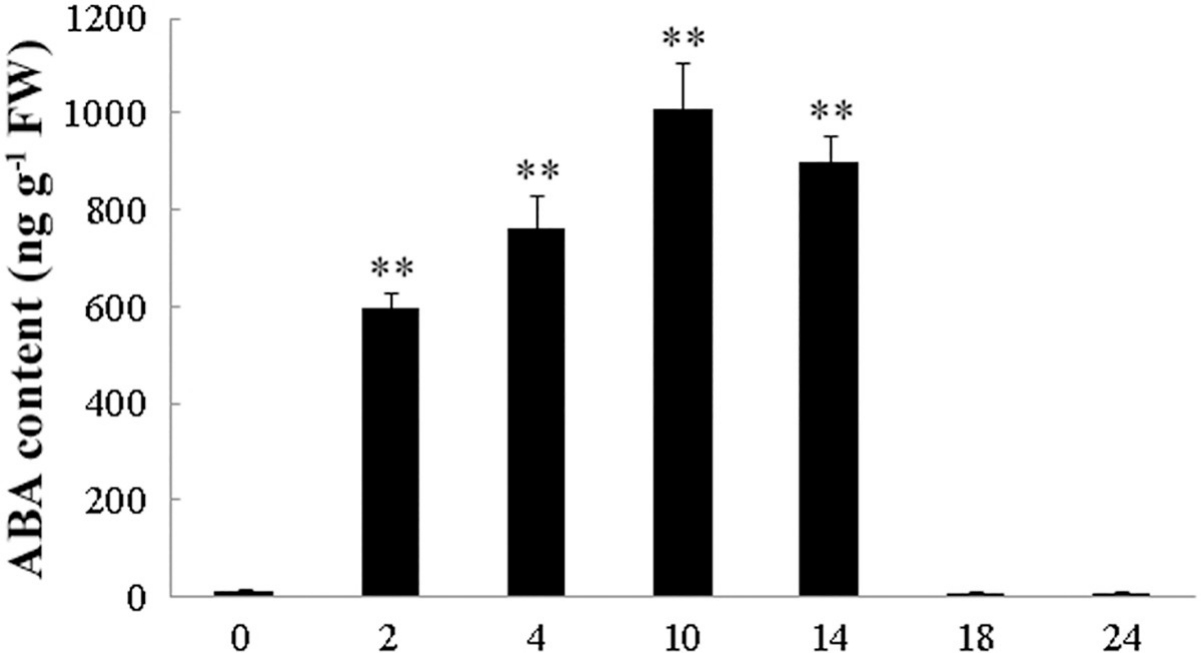
The ABA level in peanut leaves in response to 0, 2, 4, 10, 14, 18, or 24 h of drought stress. The ABA levels in peanut leaves at the presence or absence of drought were determined triplicately for each sample (The ABA contents in peanut leaves during control conditions showed no obvious difference with that in 0 h stressed sample and were not presented). All data are presented as mean ± standard errors (SE) of three replicates. The asterisk above each bar indicates a significant difference between stressed and controlled samples at *P* < 0.01 (**).

In the present study, the ABA level in peanut leaves in response to 0, 2, 4, 10, 14, 18, or 24 h of drought stress was respectively determined. As shown in Fig 12, the ABA level was significantly increased by drought stress. The ABA content rapidly began to accumulate within 2 h (a 56.6-fold increase) from the start of stress. The highly and rapidly stress up-regulated expressions of genes involved in ABA production and transport, particularly *AhNCED1* (Fig 8B), *AhBG11* and *AhBG24* (Fig 9), and *AhABCG22*.*1* and *AhABCG22*.*2* (Fig 11), might contribute to the rapid ABA accumulation (Fig 12).

At 10 h of drought stress, the ABA level reached a peak, 95.9 times higher than that in the control (Fig 12). The ABA content then started to decrease at 18 h of the stress, and reduced to an even lower level than that of the normal (likely due to severe damages induced by drought stress) (Fig 12). ABA homeostasis maintained through a balance between the production, catabolism and transport, rather than simply by the biosynthesis. Consistent with this idea, the expressions of genes involved in ABA production (*AhZEP*, *AhNCED1*, *AhABA3*, *AhAAO2*, *AhBG12* and *AhBG24*) (Figs 8 and 9), catabolism (*AhCYP707A3* and *AhUGT71K1*) (Fig 10), and transport (*AhABCG22*.*1* and *AhABCG22*.*2*) (Fig 11) were all up-regulated upon drought stress, although the induction of biosynthetic gene (*AhNCED1*) (Fig 8) was much higher than that of catabolic genes (*AhCYP707A3* and *AhUGT71K1*) (Fig 10). This difference in induction kinetics of gene expression may define the significant accumulation of stress-induced ABA levels (Fig 12).

## Conclusions

The two ABA-producing pathways, taking place in different compartments, coordinate to maintain the cellular ABA levels. Additionally, the catabolic pathways play a critical role in the regulation of cellular ABA levels. Furthermore, the PM-localized ABA-specific transporters also contribute to the regulation of cellular ABA levels in plant cells. The differential subcellular localization of all the key enzymes involved in ABA metabolism and transport indicates that integrated regulatory networks involving multiple organelles are implicated in the regulation of cellular ABA homeostasis. Identification of the components involved in the regulation of ABA homeostasis, including those that function in production and catabolism, as well as in transport between compartments, helps to understand the regulatory networks at the molecular level. Here we demonstrate that, in response to drought stress, ABA accumulation levels in peanut leaves agree well with the up-regulated expressions of ABA-producing genes and PM-localized ABA importer genes, although the expressions of ABA catabolic genes also increase, suggesting that ABA homeostasis in peanut leaves in response to drought may be coordinated by a master regulatory circuit that involves production, catabolism, and as well as transport.
